# Whole-Brain CT Perfusion and CT Angiography Assessment of Moyamoya Disease before and after Surgical Revascularization: Preliminary Study with 256-Slice CT

**DOI:** 10.1371/journal.pone.0057595

**Published:** 2013-02-22

**Authors:** Jun Zhang, Jianhong Wang, Daoying Geng, Yuxin Li, Donglei Song, Yuxiang Gu

**Affiliations:** 1 Department of Radiology, Huashan Hospital, Fudan University, Shanghai, China; 2 Department of Neurology, Huashan Hospital, Fudan University, Shanghai, China; 3 Department of Neurosurgery, Huashan Hospital, Fudan University, Shanghai, China; University of Münster, Germany

## Abstract

**Background/Aims:**

The 256-slice CT enables the entire brain to be scanned in a single examination. We evaluated the application of 256-slice whole-brain CT perfusion (CTP) in determining graft patency as well as investigating cerebral hemodynamic changes in Moyamoya disease before and after surgical revascularization.

**Methods:**

Thirty-nine cases of Moyamoya disease were evaluated before and after surgical revascularization with 256-slice CT. Whole-brain perfusion images and dynamic 3D CT angiographic images generated from perfusion source data were obtained in all patients. Cerebral blood flow (CBF), cerebral blood volume (CBV), time to peak (TTP) and mean transit time (MTT) of one hemisphere in the region of middle cerebral artery (MCA) distribution and contralateral mirroring areas were measured. Relative CTP values (rCBF, rCBV, rTTP, rMTT) were also obtained. Differences in pre- and post- operation perfusion CT values were assessed with paired *t* test or matched-pairs signed-ranks test.

**Results:**

Preoperative CBF, MTT and TTP of potential surgical side were significantly different from those of contralateral side (*P*<0.01 for all). All graft patencies were displayed using the 3D-CTA images. Postoperative CBF, rCBF and rCBV values of surgical side in the region of MCA were significantly higher than those before operation (*P*<0.01 for all). Postoperative MTT, TTP, rMTT and rTTP values of the surgical side in the region of MCA were significantly lower than those before operation (*P*<0.05 for all).

**Conclusion:**

The 256-slice whole-brain CTP can be used to evaluate cerebral hemodynamic changes in Moyamoya disease before and after surgery and the 3D-CTA is useful for assessing the abnormalities of intracranial arteries and graft patencies.

## Introduction

Moyamoya disease is characterized by a chronic progressive steno-occlusive vasculopathy affecting the terminal internal carotid arteries [1]. Characteristic radiographic findings confirm the diagnosis, and recognition of the disease early in its course together with prompt institution of therapy, are critical in order to achieve the best outcome in patients. Revascularization surgery appears to be effective in preventing stroke in patients with Moyamoya [2]. The clinical presentation and outcome of Moyamoya disease remain varied based on angiographic studies alone; otherwise there are intrinsic limitations of angiographic studies used to evaluate the complex interplay of cerebral circulation in qualitative and quantitative methods. Brain perfusion data plays an important role in the preoperative pathologic assessment of patients with Moyamoya disease and in the therapeutic planning. Compared to PET, SPECT and MRI, the advantages of CT perfusion (CTP) include its high accessibility, the fast speed of the examination, and the lack of significant contraindications. The isotropic volume data of the whole-brain can be acquired over a 12-cm area once without helical scanning with a 256-slice CT scanner. Thus, perfusion data of the entire brain can be obtained by means of continuous dynamic scanning; allowing evaluation of whole-brain perfusion [3], and dynamic whole-brain CT angiographic images can be obtained from the arterial phase to the venous phase in the same examination. The purpose of the present study was to evaluate the value of the application of 256-slice whole-brain CTP in confirming the graft patency with vessel images obtained in the source data of CTP as well as investigating cerebral hemodynamic changes in Moyamoya disease before and after surgical revascularization.

## Materials and Methods

### Subjects

This study was approved by the local ethical committee (Institutional Review Board of Huashan Hospital, Fudan University), and written informed consent forms were obtained directly from patients or the next of kin. From August 2009 to October 2010, 39 subjects (16 men, 23 women; age range,16–62 years; mean age, 40.8 years) with Moyamoya disease confirmed by DSA according to Suzuki's (1969) criterion were included. 39 patients with Moyamoya disease including 30 cases of ischemic-type and 9 cases of hemorrhagic-type were studied before surgery and after surgery. The surgical treatment procedures of direct superficial temporal artery-middle cerebral artery (STA-MCA) bypass combined with encephaloduromyosynangiosis were performed in the side of ischemic hemisphere. The patency of the anastomosis was verified using intraoperative ultrasonic Doppler.

### CT Scanning and Image Processing

The imaging protocol consisted of whole brain CTP, dynamic 3D CT angiography (CTA) before and after surgery. All subjects were evaluated with 256-slice CT. CTP studies were performed in the transverse plane by using a 256-slice CT scanner (Brilliance iCT; Philips Medical Systems, Cleveland, Ohio, USA) before and after surgical revascularization. A 50-ml bolus of nonionic contrast media (Omnipaque, iodine 350 mgI/ml; GE Healthcare, Shanghai, CN) was administered into an antecubital vein by using a power injector (Stellant Injection System; Indianola, PA, USA) with an injection rate of 5 ml/s. CT scanning was initiated 5 seconds after the start of the injection with the following acquisition parameters: JOG mode(back and forth toggle move of the table),120 kV tube voltage,125 mAs,5 mm slice thickness,128×0.625 mm collimation,0.4 s rotation time,1.9 s cycle time, 22-cm field-of-view (FOV),512×512 image matrix size, 24 slices. Helical scanning was not performed. A total of 312 slices were obtained with a scan time of about 50 seconds and 120 mm scan length. Brain standard reconstruction was performed with the CT system. The gantry angle was parallel to and above the orbital roof to avoid radiation exposure to the lens.

### Post-processing of CT Data

**Figure 1:**
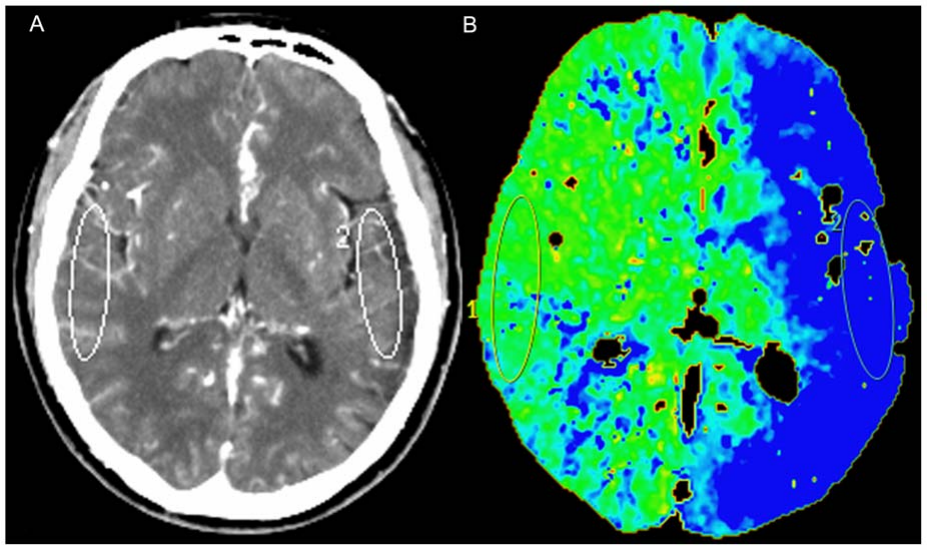
Regions of interest (ROIs) were placed on cortical regions in the middle cerebral artery territory. ROIs were drew in (**A**) the reference CT image, and (**B**) vessel-removed mean transit time (MTT) map.

The whole-brain plain images, dynamic 3D-CTA and whole-brain perfusion images were reconstructed from source data acquired with dynamic scans. The dynamic 3D-CT angiographic images were generated from volumetric data acquired in the arterial, capillary, and venous phases of CTP source data. The 3D volume-rendered images of cerebral arteries performed at the peak of the arterial time-attenuation curve were the same as conventional CTA images using advanced vessel analysis software installed in the CT workstation. CTP data were analyzed using brain perfusion software (Extended Brilliance Workstation v 3.0, Philips Medical Systems). The software relies on the central volume principle to calculate perfusion parameters from the time-concentration curve. The software applies curve fitting by a least-mean squares method to obtain mathematical descriptions of the time-attenuation curves, and the mean transit time (MTT) map was calculated by a closed-form (noniterative) deconvolution operation from the time-concentration curve of a particular voxel and the arterial input function (AIF). The first artery to reach peak enhancement on the time-attenuation curve was selected as the AIF. The vein with the largest area under the time-attenuation curve was selected as the venous outflow function. For each voxel, the cerebral blood volume (CBV) map was calculated from the areas under the time-concentration curves. The cerebral blood flow (CBF) map for each voxel was finally calculated according to the following equation combining the CBV and MTT value: CBF = CBV/MTT. Large cortical vessels were automatically excluded via brain perfusion software. Two experienced neuroradiologists independently drew 2 standardized elliptical mirrored regions of interest (ROIs) manually on the basal ganglia (BG) section level of the reference CT image (Figure 1) over the cortical gray matter of MCA territory. Perfusion CT absolute values of one hemisphere in the region of MCA distribution and contralateral mirroring areas in functional maps were measured; from each ROI, CBF, CBV, MTT and time to peak (TTP) were calculated. The lower CTP absolute values have a role for planning the treatment strategy of the surgical side in this study. The relative cerebral blood flow (rCBF), relative cerebral blood volume (rCBV), relative time to peak (rTTP), and relative mean transit time (rMTT) were obtained as follow: relative CTP values = absolute CTP values _surgical side_/absolute CTP values _contralateral side_.

### Statistical Analysis

For CTP parameters, the mean of ROI values on each ipsilateral and mirrored contralateral hemisphere was calculated before and after surgery. The preoperative mean ROI values between ipsilateral and mirrored contralateral and mean ROI values in surgical side between pre-surgery and post-surgery were compared in all 39 cases. Normal distribution data were expressed as mean±SD while skew distribution of measurement data were expressed as median (P_25_–P_75_). Differences in pre- and post- operation perfusion CT values were assessed using paired *t* test and *t* values were obtained, while skew distribution of measurement data were assessed with matched-pairs signed-ranks test and *z* values were derived. Values with *P*<0.05 were considered statistically significant. All raw data were analyzed using statistical software (Intercooled, version 7.0, 2001; Stata, College Station, Tex; Windows Office Excel 2003, Microsoft, Redmond, Wash).

## Results

**Figure 2:**
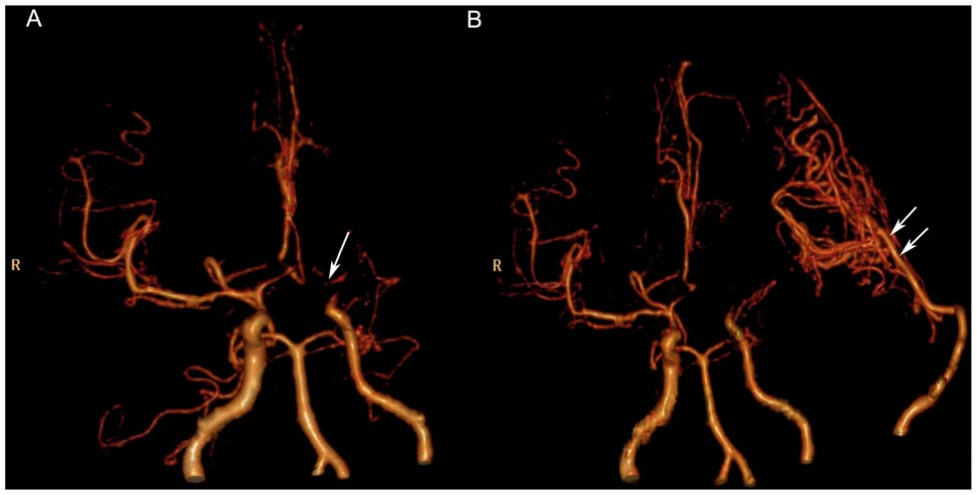
CT Angiography (CTA) images in frontal projection before and after bypass in Moyamoya disease. (**A**) The preoperative volumetric CTA acquired in the arterial phase of whole-brain CT perfusion (CTP) shows the left internal carotid artery (ICA) is occluded in the supraclinoid portion, and “Moyamoya vessels”(arrow) can be seen in the cerebral base. The right supraclinoid ICA and the proximal anterior cerebral artery (ACA) and middle cerebral artery (MCA) are markedly stenosed. (**B**) The arrows indicate the left-side direct graft patency with collateral vessels from the left external carotid artery to the MCA territory on the postoperative volumetric CTA acquired in the arterial phase of whole-brain CTP.

There were abnormal densities in brain tissue in most of patients, among which 33 cases had brain infarction including 8 cases of brain hemorrhage with encephalomalacia and 2 cases of atrophy of brain. Normal density was seen in 6 cases. The stenosis and occlusion of multiple cerebral vessels could be well demonstrated on volumetric CTA (Figure 2A). All the direct graft patencies were displayed by volumetric CTA in the arterial phases of CTP source data (Figure 2B).

**Figure 3:**
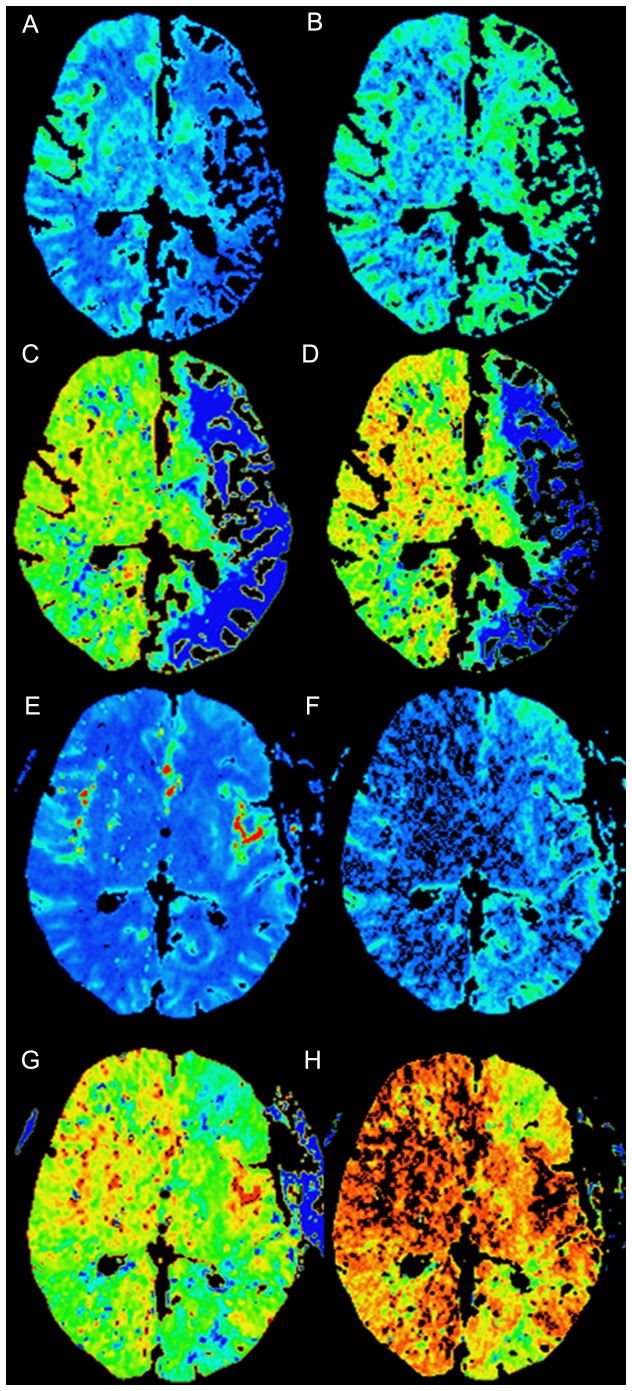
CT perfusion images before and after surgery. Images show cerebral hemodynamics changes of pre- and post-operation of a left-sided superficial temporal artery-to-middle cerebral artery anastomosis in Moyamoya disease. Axial perfusion CT images show reduced (**A**) cerebral blood flow (CBF), increased (**B**) cerebral blood volume (CBV), delayed (**C**) mean transit time (MTT), and (**D**) time to peak (TTP) in the region of left middle cerebral artery before surgery. The postoperative whole-brain perfusion CT images show increased (**E**) CBF, reduced (**F**) CBV, (**G**) MTT and (**H**) TTP in the region of left middle cerebral artery.

Preoperative CBF, MTT and TTP of potential surgical side were significantly different from those of contralateral side (*z* = −4.58, *z* = 4.26, *t* = 3.30, respectively; *P*<0.01 for all) (Table 1). The CBF in the affected regions significantly improved immediately after operation (Figure 3). Postoperative CBF, rCBF and rCBV values of surgical side were significantly higher than those before operation (*z* = −3.92, *z* = −4.81, *z* = −3.21, respectively; *P*<0.01 for all). Postoperative MTT, TTP, rMTT and rTTP values of surgical side were significantly lower than those before operation (*z* = 2.87,*P* = 0.0041;*z* = 2.49,*P* = 0.0126;*z* = 3.24,*P* = 0.0012;*z* = 4.08,*P* = 0.0000;respectively) (Table 2). However, no significant difference was detected for changes of CBV after revascularization (*P*>0.05).

**Table 1:** Comparison of Preoperative Perfusion CT Values of Potential Surgical Side in the Region of Middle Cerebral Artery and Those of Contralateral Side [mean±SD; median (P_25_–P_75_)].

Perfusion CT Values	Potential surgical side	Contralateral side	*z*/*t*	*P*
CBF (ml·100 g^−1^·min^−1^)	33.45(24.75–48.85)	50.47(34.8–96.82)	−4.58*	0.0000
CBV (ml·100 g^−1^)	3.68±1.90	3.80±1.92	−0.74**	0.4648
MTT(s)	5.42(4.62–7.85)	3.84(2.08–5.97)	4.26*	0.0000
TTP(s)	20.23±4.17	18.58±3.62	3.30**	0.0021

CBF = cerebral blood flow, CBV = cerebral blood volume, MTT = mean transit time TTP = time to peak. *P*<0.05 was considered statistically significant. Normal distribution data were expressed as mean±SD while skew distribution of measurement data were expressed as median (P_25_–P_75_).* *z* value was derived by matched-pairs signed-ranks test, and ** *t* value was derived using paired *t* test.

**Table 2:** Comparison of Perfusion CT Values of Surgical Side in the Region of Middle Cerebral Artery Pre- and Post-operation [mean±SD; median (P_25_–P_75_)].

Perfusion CT Values	Pre-operation	Post-operation	*z*	*P*
CBF (ml·100 g^−1^·min^−1^)	33.45(24.75–48.85)	57.99(34.17–86.21)	−3.92	0.0001
CBV (ml·100 g^−1^)	3.68±1.90	2.56(1.96–5.88)	−0.30	0.7662
MTT(s)	5.42(4.62–7.85)	3.71(2.05–5.04)	2.87	0.0041
TTP(s)	20.23±4.17	17.73(16.24–20.77)	2.49	0.0126
rCBF	0.72(0.42–0.94)	1.29(1.12–1.82)	−4.81	0.0000
rCBV	0.94(0.8–1.14)	1.23(0.84–1.86)	−3.21	0.0013
rMTT	1.18(1.04–1.96)	0.95(0.77–1.30)	3.24	0.0012
rTTP	1.03(1.00–1.15)	0.99(0.95–1.02)	4.08	0.0000

CBF = cerebral blood flow, CBV = cerebral blood volume, MTT = mean transit time TTP = time to peak, rCBF = relative cerebral blood flow, rCBV = relative cerebral blood volume, rMTT = relative mean transit time, rTTP = relative time to peak. *P*<0.05 was considered statistically significant. Normal distribution data were expressed as mean±SD while skew distribution of measurement data were expressed as median (P_25_–P_75_).

## Discussion

Moyamoya disease is a cerebral arterial angiopathy characterized by chronically progressive stenosis and occlusion of the intracranial internal carotid arteries and their proximal branches together with the formation of collateral vessel [1]. Symptoms of cerebral ischemia in Moyamoya are typically associated with the regions of the brain supplied by the internal carotid arteries and middle cerebral arteries. The natural history of Moyamoya is variable. Disease progression can be slow, with rare, intermittent events, or fulminant, with rapid neurologic decline [4].

Studies have reported early diagnosis of Moyamoya coupled with the expeditious institution of therapy is of paramount importance. Revascularization surgery appears to be effective in preventing stroke in patients with Moyamoya disease [2]. In order to identify patients who suffer from hemodynamic cerebral insufficiency and can benefit from cerebral revascularization procedures, CT scanning has been established to reliably and accurately measure the critical cerebrovascular reserve capacity [5].

Many techniques such as conventional angiography, transcranial Doppler, MRI, CTA, CTP, Xe-CT, PET, and SPECT with acetazolamide challenge have all been used in the evaluation of patients with Moyamoya. However, each technique has its own advantages and drawbacks. In terms of evaluating hemodynamic status in Moyamoya disease, SPECT has been considered a reference standard [6]. However, SPECT has to be performed usually in a 2-day setting due to tracer kinetics. Moreover, it provides less morphologic information than CT or MR imaging. CTP is a more readily accessible method for the evaluation of cerebral perfusion than SPECT [7], [8]. With contrast to other methods, perfusion CT can rapidly detect the size of hypoperfused regions in the setting of acute stroke and lack of significant contraindications. Except for the radiation dose and the fact that iodinated contrast material, it may be unsuitable for some patients [9]. The most common imaging technique for the primary diagnostics of hemispheric infarcts and hemorrhage is brain CT. CT angiography could be easily performed in the same session for these patients at considerably lower cost compared with MR angiography, and without transfer of the patient to another imaging unit. Especially, the short duration of CTP examinations make CTP an ideal technique for postsurgical patients without artifacts of movement, susceptibility and hemorrhage. For MRI perfusion, the quantification of CBF is more complex with MRI perfusion than with CTP because the relationship between the signal intensity and the gadolinium concentration is not always linear [10].

The 256-slice CT, which enables the entire brain to be scanned in a single examination with administration of one contrast medium bolus, does not have the spatial coverage limitations that other multi-detector row CT systems possess and can be used to obtain the whole-brain perfusion data. Meanwhile, in our study, the whole-brain dynamic 3D-CTA images were reconstructed from source data acquired with dynamic scans. Both quantitative method of measuring cerebral hemodynamic and CTA images were performed in one examination. Whole brain perfusion imaging can be performed to ascertain the occurrence and the extent of ischemia, and CTA can be performed to investigate the morphologic status of cerebral arteries. Although cerebral angiography is considered the 'gold standard' imaging modality for diagnosis and postoperative follow-up of Moyamoya disease, its invasive nature has a number of limitations including procedural risks, costs, and limited availability that makes it less desirable than CTA for screening for intracranial stenosis and occlusion or following patients with these conditions. CTA has relatively fewer risks, costs less, is more readily available and appears to be highly accurate, suggesting that this approach may be preferred for identifying patients with intracranial stenosis and occlusion [11]. Owing to the risks associated with angiography, alternatives such as the use of noninvasive imaging have been proposed. In this study, CTA need not been performed in fact, it was additionally reconstructed from CTP source data. Moyamoya is also best detected by means of CTA. Characteristic CT angiographic findings confirm its diagnosis. All graft patencies were displayed by volumetric CTA. With combination of CTA and CTP in one study, the volume of contrast medium administered to the patient is reduced, which is of great benefit to pediatric patients, elderly patients, and patients with renal insufficiency.

Patients with Moyamoya disease have reduced CBF and increased CBV, MTT and TTP. These hemodynamic changes in Moyamoya disease are explained by decreases in the cerebral perfusion pressure and associated vasodilation [12]. The assessment of cerebral ischemia by means of perfusion parameters derived from perfusion CT provides valuable information to predict outcome. Images of CBF, CBV, TTP and MTT are interpreted together on a workstation permitting the use of visual assessment combined with quantitative analysis with ROI. Regions of decreased perfusion are often represented as regions of prolonged TTP and MTT. TTP and MTT maps have the capability of being quite sensitive to the presence of altered brain perfusion. However, the calculation of accurate absolute values is impractical, and the use of absolute thresholds for therapeutic decision making is questionable [13]. Comparing CBF, CBV, TTP and MTT values between abnormal regions and mirror-image control regions is an effective method of measuring the degree of under perfusion present in a given case or location. In the present study, rCBF and rCBV values were found to be significantly increased, rMTT and rTTP values were found to be significantly reduced after revascularization surgery. It appears that these phenomena are probably due to rapid parenchymal perfusion after revascularization surgery and decrease of collateral vessel formation.

There were limitations in the present study. First, the radiation exposure is unavoidable in perfusion CT, the radiation dose is increased because of repeat scans. Further research is needed to determine whether the radiation dose can be reduced. Recently, an attempt was made to reduce the radiation dose using iDose technology with relatively low-dose conditions [14]. In one whole-brain CT perfusion case performed at our hospital, the dose-reduction method used allowed the effective dose to be reduced half, which was comparable to the total dose for conventional perfusion CT, CTA, and unenhanced head CT.

The second limitation concerns AIF. In the CBF calculations with CT perfusion imaging, the choice of a reference artery is critical. An artery of the anterior circulation such as anterior cerebral artery (ACA) or MCA is usually selected as reference artery automatically. However, in a patient with Moyamoya disease, ACAs and/or MCAs might be occluded. Therefore, we propose that it is optimal to select basilar artery or posterior cerebral artery (PCA) as reference artery manually, because PCA is hardly affected by revascularization surgery [15].

The third limitation concerns the accurate drawing of MCA territories before and after revascularization surgery. Drawing the same level and size of ROI was challenging [16], [17]; therefore, we measured perfusion changes between abnormal regions and mirror-image control regions, and the relative perfusion values were compared before and after revascularization surgery to minimize individual variability. However, potential bias might have been introduced by the change of brain volume caused by craniotomy in ROIs.

## Conclusions

The 256-slice whole-brain CTP and 4D-CTA have the potential for the non-invasive assessment of the abnormalities of intracranial arteries, the graft patency and cerebral perfusion changes in Moyamoya disease before and after surgery. The postoperative volumetric CTA acquired in the arterial phase of whole-brain CTP could display the graft patencies instead of conventional CTA; meanwhile reducing the radiation exposure in a single examination.
